# A profile of traumatic brain injuries and associated cervical spine injuries at a regional hospital in the KwaZulu-Natal Province

**DOI:** 10.4102/safp.v62i1.5136

**Published:** 2020-10-08

**Authors:** Maamei L. Malale, Nicholas Dufourq, Nivisha Parag

**Affiliations:** 1 Department of Emergency Medicine, Nelson Rolihlahla School of Medicine, University of KwaZulu-Natal, Empangeni, South Africa

**Keywords:** traumatic brain injuries, cervical spine injuries, combined diagnosis, trauma, rural medicine

## Abstract

**Background:**

Clearing the cervical spine in an unconscious blunt trauma patient is an elusive concept. The aim of this study was to describe the incidence of cervical spine injury (CSI) in patients with a traumatic brain injury (TBI). The study was conducted on patients who underwent imaging of both the cervical spine and the brain in one sitting at a busy government healthcare facility in Pietermaritzburg.

**Methods:**

This was a retrospective, cross sectional study of all the trauma patients presenting to a regional hospital emergency department (ED) in the KwaZulu-Natal (KZN) Province, who underwent computed tomography (CT) imaging of the brain and the cervical spine in one sitting during the period January 2016 to June 2016.

**Results:**

Adult males formed the majority (78.9%) of the study population and had the highest incidence of TBI, the most common identified pathology in CT being parenchymal injuries (41%). The mechanisms that resulted in the majority of injuries sustained were assault (38.7%) and motor vehicle collisions (MVCs) (25%), while seven patients (4.76%) had a combined diagnosis of TBI and CSI. The average Glasgow Coma Scale (GCS) was 12.

**Conclusion:**

Young adult males are at the greatest risk of sustaining TBI, with assault being the most common mechanism of injury. Combined diagnoses of TBI and CSI are rare and were mostly noted in patients involved in MVCs and pedestrian vehicle collisions. While the chance of an abnormal CT scan increased with a decreasing GCS score, 33% of patients with a mild TBI did not have abnormal CT findings, and 25% patients with severe TBI had no abnormal CT findings.

## Introduction

Trauma continues to be a major cause of morbidity and mortality worldwide, with the World Health Organisation (WHO) estimating in 2012 that trauma-related deaths caused 9% of global mortalities.^1^ A traumatic brain injury (TBI) is defined as:

[*A*] nondegenerative, non-congenital insult to the brain from an external mechanical force, possibly leading to permanent or temporary impairment of cognitive, physical and psychosocial functions, with an associated diminished or altered state of consciousness.^2^

Traumatic brain injury is most frequently classified into three distinct categories, that is, mild (13–15), moderate (9–12) and severe (< 8), according to the Glasgow Coma Scale (GCS), which is a neurological scale that was designed to objectively assess impaired consciousness.^3^ In addition, Advanced Trauma Life Support (ATLS) teaches that a GCS less than 8 necessitates intubation and this is applied to South African trauma facilities. The evidence, which is supported by clinical decision-making rules, suggests that if a patient has a decreased level of consciousness, a cervical spine injury (CSI) cannot be ruled out and needs to be investigated. Severe CSIs are of particular concern regarding trauma, as they carry a high morbidity and are often associated with TBI, both being reported in 74% of patients.^4^

The United States of America’s Centre for Disease Control and Prevention reported a 53% increase in emergency department (ED) visits, hospitalizations and deaths associated with TBI in 2014 compared to 2006.^5^ The Statistics South Africa 2016 report noted that non-natural causes of death accounted for 11.2% of all deaths in 2014, with two of the most common causes being assault and motor vehicle collisions (MVCs).^6^ Males were more likely to be assaulted, while females were often involved in MVCs.^6^ In 2014, trauma contributed to 27% of ED visits in the KwaZulu-Natal (KZN) Province, South Africa, of which 33% were due to MVCs, the remainder being a result of assaults and other external causes of accidental injury.^7^

In a trauma mortality audit conducted in KZN in 2010 and 2011, the three leading causes of trauma-related deaths were head injuries (32.6%), polytrauma (29.7%), mainly due to blunt trauma caused by MVCs and pedestrian vehicle collisions (PVCs), and chest trauma sustained as a result of a penetrating trauma (27.4%).^8^ The most frequent mechanism of injury associated with TBI in a study done in Pietermaritzburg (KZN) in 2014 was interpersonal violence (39.4%), with a male to female ratio of 4.7:1.^9^ A study in Toronto, Canada, in 2014 reported that the most frequent mechanism of injury in adults with combined brain and cervical spine injuries was MVC (44.3%).^10^ In the USA, the elderly were most likely to sustain injuries due to falls, while young males sustained injuries associated with MVCs.^11^

Fujii et al. estimated that, as a percentage of all trauma, the incidence of combined TBI and CSI was between 1.7% and 8%^12^ in the USA. They reported having challenges diagnosing CSI in patients with TBI with associated risk factors, such as hypotension, due to multiple injuries.^12^ Several clinical decision rules have been validated to decrease the probability of missing a combined TBI and CSI, and have been widely applied to trauma patients presenting to resource-limited settings, including EDs in South Africa.^13,14^ As investigations such as computed tomography (CT) imaging are expensive, they should only be used when indicated in resource-constrained settings, such as South Africa. While the literature shows that combined diagnosis of TBI and CSI in South Africa is rare, the current guidelines dictate that a patient with blunt trauma and a decreased level of consciousness necessitates CT imaging of both the brain and the cervical spine. A CSI can be potentially catastrophic if missed, the estimated missed rate after blunt injury being 2.4%.^15^

Edendale Hospital is a peri-urban regional level public sector facility in Pietermaritzburg with 900 beds and serves a population of approximately 1.4 million people. The ED is estimated to see an average of 2398 patients per month with a peak of approximately 2897 during the festive season. This hospital services five district hospitals and 18 clinics, with resources and expertise ranging from on-site trauma intervention to CT imaging. Injury from interpersonal violence is one of the major health burdens in this region, with an annual incidence of approximately 16 682 patients presenting with trauma, of whom 2621 (15.7%) are orange and red codes (life-threatening), according to the South African Triage Score (SATS). In 2016, the records in the radiology department of Edendale Hospital indicated that approximately 400 combined brain and cervical spine imaging scans were performed. From January 2016 to June 2016, the radiology department performed 421 computed tomography of the brain (CTB) scans, of which 160 were combined brain and cervical spine scans. A review indicated that 13 injuries were initially thought to be due to trauma but were medical and therefore excluded, as were the 65 that were trauma CTB without CT of cervical spine; the indications for these were mainly facial injuries.

Edendale Hospital’s radiology department is staffed with medical officers and consultants during the day, with limited radiology cover after-hours. Patients seen at the hospital for TBI are discussed with the neurosurgery department of the provincial level Inkosi Albert Luthuli Central Hospital for possible transfer and further management. The NEXUS and Canadian C-Spine rules, which have been validated for screening clinically significant CSI after blunt trauma, are used at Edendale to risk stratify and justify the need to image the cervical spine and the brain.^16^ However, they were developed mainly for MVC and PVC, respectively. patients, their relevance to local conditions having not been established in terms of the nature of trauma being seen in the Edendale ED.

Little research has been done in South Africa on the extent of combined diagnosis of TBI and CSI in case of traumatic injuries. The aim of this study was therefore to describe the incidence of CSI in patients with a TBI, among those who underwent imaging of both the cervical spine and the brain in one sitting, at a busy government healthcare facility in Pietermaritzburg. The objectives of this study were to describe the demographic and clinical profile of patients with CSI and TBI, the radiological findings of these injuries on CT imaging, the incidence of TBI with concurrent CSI and the mechanisms of these injuries.

## Methods

This was a retrospective chart review conducted at Edendale Hospital of all trauma patients presenting to the ED requiring CT imaging of the brain and cervical spine (as a result of suspected brain and cervical spine injuries) during the period 01 January to 30 June 2016. Trauma patients who underwent imaging of the brain only were excluded, as there was no way of ascertaining a CSI. CT imaging was interpreted by the radiology medical officers and consultants, and formal reports printed out and placed in the patient’s files.

The medical records of those patients who underwent imaging of both the cervical spine and the brain were included, the data being entered onto a database for analyses. For the descriptive statistics, Statistical Product and Service Solutions (SPSS) version 16.0 Inc. was used, with the statistical significance between the independent variables (male, female, age groups, types of injuries, mechanisms of injury, GCS, intubated or not) being calculated by the Pearson chi square test, and a *p*-value less than 0.05 was considered significant.

### Ethical consideration

Ethical approval was granted by the Biomedical Research Ethics Council of the Faculty of Health Sciences of the University of KwaZulu-Natal (reference number BE042/17) and the study was registered with the KZN Department of Health database (KZ_2016RP45_185).

## Results

During the study period, 147 patients were identified and included in the data analysis, and divided into subgroups according to the five WHO age categories for data interpretation.^17^ Table 1 shows that in terms of the demographic and clinical profile of those experiencing a trauma, most were male in the young adult and adult age groups. Adults were generally involved in MVCs and children under 10 years in PVCs, whereas the elderly often sustained falls. Men were more likely to get injured (*p* = 0.033) but more women were intubated than men (*p* = 0.014); this may mean women presented a more severe decrease in the level of consciousness than men did.

**TABLE 1:** Patient demographics according to age category (*n* = 147).

Variable	Characteristic	Age categories according to the WHO^17^									
		Child: 0 to 9		Adolescent: 10 to 19		Young adult: 20 to 24		Adult: 25 to 59		Elderly: over 60	
		Number	%	Number	%	Number	%	Number	%	Number	%
Description	Total	18	12.0	13	9.0	21	14.0	87	59.0	8	5.0
Gender	Male	9	50.0	6	46.0	16	76.0	77	89.0	8	100.0
	Female	9	50.0	7	54.0	5	24.0	10	11.0	0	-
Mechanism of injury	Assault	2	3.5	3	5.3	12	21.1	37	64.9	3	5.3
	MVC	4	10.8	5	13.5	3	8.1	25	67.6	0	-
	PVC	7	23.3	4	13.3	4	13.3	14	46.7	1	3.3
	Fall	2	22.2	0	-	1	11.1	3	33.3	3	33.3
	Animal-related	3	60.0	1	20.0	0	-	1	20.0	0	-
	Unknown	0	-	0	-	1	11.1	7	77.8	1	11.1
GCS category	Mild (13–15)	13	14.8	8	9.1	13	14.8	49	55.7	5	5.7
	Moderate (9–12)	3	8.6	3	8.6	6	17.1	22	62.9	1	2.9
	Severe (< 8)	2	8.3	2	8.3	2	8.3	16	66.7	2	8.3
Intubation	Yes	1	3.7	2	7.4	4	14.8	18	66.7	2	7.4
	No	17	14.2	11	9.2	17	14.2	69	57.5	6	5.0
CTB findings	Extradural	1	5.0	1	5.0	5	25.0	12	60.0	1	5.0
	Subdural	0	-	0	-	2	16.7	10	83.3	0	-
	Parenchymal	2	3.44	7	12.09	12	20.68	34	58.6	3	5.17
	SAH	4	11.4	2	5.7	5	14.3	23	65.7	1	2.9
	IVH	0	-	3	50.0	0	-	2	33.3	1	16.7
	Tonsillar Herniation	0	-	0	-	0	-	2	66.7	1	33.3
	Pneumocephalus	0	-	0	-	2	33.3	4	66.7	0	-
Cervical spine	No Pathology	17	12.4	13	9.5	20	14.6	79	57.7	8	5.8
	Level 1–3	0	0.0	0	0.0	0	0.0	2	100.0	0	0.0
	Level 4–5	0	0.0	0	0.0	0	0.0	2	100.0	0	0.0
	Level 6–T1	1	16.7	0	0.0	1	16.7	4	66.7	0	0.0
	Total	18	12.2	13	8.8	21	14.3	87	59.2	8	5.8

MVC, motor vehicle collision; PVC, pedestrian vehicle collision; GCS, Glasgow Coma Scale; SAH, subarachnoid haemorrhage; IVH, intraventricular haemorrhage.

One patient sustained two vertebral body injuries; therefore, the total is 11 injuries.

In terms of the clinical profile, Table 2 shows that the chance of abnormal CT findings increases with a decreasing GCS. Patients with a moderate TBI have a greater chance of an abnormal finding than patients with a mild TBI (OR 3.4 95% CI: 1.2–7.7). Patients with a severe TBI have a greater chance of an abnormal finding than patients with a mild TBI (OR 6.1 95% CI: 2.2–17.0). The GCS does not predict the number of intracranial pathologies, merely the probability of the presence or absence thereof (*p* = 0.37).

**TABLE 2:** Severity of brain injury.

GCS	No findings		Any findings		Total		OR	95% CI	*p*
	*N*	%	*N*	%	*N*	%			
Mild	59	67	29	33.0	88	100	Reference	-	-
Moderate	13	37	22	62.9	35	100	3.4	1.2–7.7	0.003
Severe	6	25	18	75.0	24	100	6.1	2.2–17.0	0.001
**Total**	**78**	**-**	**69**	**-**	**147**	**-**	**-**	**-**	**-**

OR, odds ratio; CI, confidence interval.

In terms of the radiological findings of these injuries on the CT, of the 147 patients, 78 had no intracranial pathology while the remaining 69 sustained a combined total of 139 injuries (Table 3). The most encountered intracranial pathology on the CT imaging was parenchymal injury, with haemorrhagic contusions being the most common finding. Subarachnoid haemorrhage and epidural haematoma were also frequently identified. The patients who were reported to have tonsillar herniation on CT had GCS ranging between 6 and 9, and two out of the three had an unknown mechanism of injury. In addition, 48 sustained more than one intracranial pathology, with the highest incidence accounted for by assault.

**TABLE 3:** Mechanisms of injury according to the number of intracranial pathologies sustained.

Method of injury	No intracranial pathology (*N* = 78) (53.1%)		One intracranial pathology (*N* = 21) (14.3%)		Two intracranial pathologies (*N* = 30) (20.4%)		Three intracranial pathologies (*N* = 14) (9.5%)		Four intracranial pathologies (*N* = 4) (2.7%)		Total (*N* = 147)
	*n*	%	*n*	%	*n*	%	*n*	%	*n*	%	
Assault	23	29.5	10	47.6	16	53.3	7	50.0	1	25.0	57
Fall	4	5.1	1	4.8	3	10.0	0	0.0	1	25.0	9
Animal-related injury	4	5.1	0	0.0	1	3.3	0	0.0	0	0.0	5
MVC	29	37.2	2	9.5	3	10.0	2	14.3	1	25.0	37
PVC	15	19.2	7	33.3	4	13.3	3	21.4	1	25.0	30
Unknown	3	3.3	1	4.8	3	10.0	2	14.3	0	0.0	9
**Total**	**78**	**100.0**	**21**	**100.0**	**30**	**100.0**	**14**	**100.0**	**4**	**100.0**	**147**

MVC, motor vehicle collision; PVC, pedestrian vehicle collision.

Regarding the radiological findings and combined diagnosis of TBI and CSI, Table 4 shows the types of injuries reported and the concurrent CSI, with 11 being identified on CT, of which 7 (63.6%) were associated with a TBI. Only one patient had neurological signs related to the CSI, this patient was found to have C7 subluxation on imaging. MVC was the common mechanism of injury that caused the CSI, followed by PVC and animal-related injuries. Reported findings included four transverse process fractures, two odontoid process fractures and one wedge fracture. Extra-axial collections (epidural and subdural haematomas) were identified in 32 patients, most of whom sustained a mild TBI. Concurrent CSIs were identified in seven patients of whom five had a mild TBI.

**TABLE 4:** Injuries sustained according to GCS category and concurrent CSI.

Glasgow Coma Scale category	Mild (13–15) (*N* = 54)		Moderate (9–12) (*N* = 44)		Severe (3–8) (*N* = 41)		Total (*N* = 147)
	*n*	%	*n*	%	*n*	%	
Subarachnoid haemorrhage	12	34.3	11	31.4	12	34.3	35
Parenchymal injury	20	35.1	22	38.6	15	26.3	57
Epidural haematoma	13	65.0	5	25	2	10	20
Subdural haematoma	4	33.3	3	25	5	41.7	12
Intraventricular haemorrhage	0	0.0	2	33.3	4	66.7	6
Tonsillar herniation	0	0.0	1	33.3	2	66.7	3
Pneumocephalus	5	83.3	0	0.0	1	16.7	6
Concurrent CSI	5	71.42	1	14.2	1	14.2	7

In terms of the mechanism of injury and severity of TBI, the majority of patients across all age groups sustained a mild TBI, while moderate and severe TBI were mostly observed in adults, with 66.7% requiring intubation (Table 5). Assault, MVCs and PVCs were the most common mechanisms of injury, while assault and PVCs were the most frequent causes of severe TBI.

**TABLE 5:** Severity of TBI according to mechanism of injury.

Mechanism of injury	GCS score						Total (*N* = 147)
	Mild (*N* = 88) (59.9%)		Moderate (*N* = 35) (23.8%)		Severe (*N* = 24) (16.3)		
	*n*	%	*n*	%	*n*	%
Assault	33	57.9	15	26.3	9	15.8	57
MVC	28	75.7	6	16.2	3	8.1	37
PVC	17	56.7	6	20.0	7	23.3	30
Fall	4	44.4	3	33.3	2	22.2	9
Animal-related injury	5	100	0	0.0	0	0.0	5
Unknown	1	11.1	5	55.6	3	33.3	9

GCS, Glasgow Coma Scale; MVC, motor vehicle collision; PVC, pedestrian vehicle collision.

## Discussion

### Traumatic brain injury and cervical spine injury

Clearing the cervical spine in a patient with a decreased level of consciousness is an elusive concept, and the literature in this regard is limited, as there have been no large prospective multicentre studies to derive a clinical decision rule for the exclusion of CSI.^16^ In South Africa, no studies have been conducted to effectively clear the cervical spine in an unconscious patient. In this study, the majority of patients that were assaulted and sustained a mild TBI did not sustain an associated CSI, similar to prior audit findings by Jerome et al. in 2014.^9^ The most common TBI was subarachnoid haemorrhage and haemorrhagic contusion. A combined diagnosis of TBI and CSI was found in 4.76% (*n* = 7) of people who underwent CT imaging of both the brain and the cervical spine at the same sitting, an incidence similar to those described by Holly et al.,^18^ who demonstrated that the most common mechanisms of injury responsible for the concurrent diagnosis of TBI and CSI were PVCs and MVCs.

### Demographics

Adult men formed the majority of the study sample and were found to have the most injuries on imaging. The ratio of males to females was 4:1, this being comparable to that described by Jerome et al. in an audit published in 2017, their ratio being 4.7:1.^9^ Men were often involved in incidents of interpersonal violence, alcohol and other substances of abuse leading to such incidents.

### The Glasgow Coma Scale

The GCS is a neurological scale used to determine the level of consciousness in a patient and consists of three aspects (motor response, eye opening and verbal response), which are then combined to give a score out of 15, with the lowest score being 3. The GCS is subclassified into mild (13–15), moderate (9–12) and severe (< 8). It is a useful tool to determine the need for radiological investigations in the form of CT imaging. There are many limitations in accurately describing the GCS in polytrauma patients, with the chances of intracranial pathology increasing with a lower score. The mechanism of injury is an important consideration when predicting the possibility of TBI and combined CSI, as was the case with a patient who sustained an animal-related injury and presented with a GCS of 15. Overall, the GCS was poorly predictive of pathology, with 78 CT brain studies showing no identified pathology, 33% of patients with mild TBI had significant intracranial pathology and 25% of patients with a severe TBI had no pathology identified on CT imaging. There are many confounders to accurately predict the probabilities of a TBI, particularly in our study, which reflected that 25% of patients with a severe TBI had no intracranial pathology. Some confounders may include alcohol intoxication, peri-orbital oedema, intubation and inter-rater variability.

### Mechanism of injury

According to a study done by Kulvatunyou et al., the incidence of CSI after blunt assault is low, and what determines the pattern and severity of injury is the fall that follows the assault.^19^ Haydel et al. reported that mortality due to MVC is greatest in the young adult group, being mainly attributed to alcohol and excessive speed.^11^ In this study, PVCs and MVCs accounted for the majority of a combined diagnosis of TBI and CSI, with other common mechanisms of injury, such as assault, not being associated with concurrent TBI and CSI. Assault had the highest incidence of all head injuries in this region, with none of these patients sustaining a CSI. However, this is a retrospective study with small numbers, which raises the question as to whether cervical spine imaging should be performed in all assaulted patients with a decreased level of consciousness. A CSI may have existed in those patients that only underwent imaging of the brain, but as the aim of the study was to establish the incidence of CSI in patients with TBI, these patients were excluded from the study.

## Limitations

This was a retrospective cross-sectional study and had a small sample size, making it difficult to arrive at conclusions, with a larger retrospective or prospective study being warranted to draw clinical significance. The sample size was determined by the amount of radiology cover available to report the CT scans. A wide range of radiology staff (grade 1 medical officers to consultants) reported the CT scans and the GCS was interpreted by a wide range of medical officers to registrars in the ED. Some patients underwent CT imaging of the brain and cervical spine at different sittings and these were also excluded, others only underwent X-ray imaging of the spine and these were unfortunately not reported at the time of the study. The findings do however highlight the need for ongoing research to refine any clinical guidelines that direct the use of expensive equipment in resource-constrained setting, such as KZN.

## Conclusion

This study showed that a combined diagnosis of TBI and CSI is relatively uncommon (4.76%). The mechanism of injury is likely to be more predictive of identifying a combined TBI and CSI diagnosis than the GCS, and a high index of suspicion for concurrent injuries should be maintained for particular injury mechanisms. It also showed that patients who were assaulted were unlikely to sustain a CSI, which could potentially decrease the volume of cervical imaging ordered on these patients. Young adult and adult males have the highest risk of a combined diagnosis, due to both the frequency of presentation and the mechanism of injury, and vigilance must be maintained in excluding a CSI, which when significant, is associated with high risks of morbidity and mortality.

## Future study

A study needs to be considered to include hospital mortality and neurological outcomes of this population that carries a high burden of disease in healthcare facilities and society in general.
